# Factors associated with help-seeking regarding sexual orientation concerns among Japanese gay and bisexual men: results from a cross-sectional survey

**DOI:** 10.1186/s13104-024-06776-x

**Published:** 2024-04-23

**Authors:** Noriyo Kaneko, Adam Orlando Hill, Satoshi Shiono

**Affiliations:** 1 Department of Global and Community Health, Graduate School of Nursing, Nagoya City University, 1-4-7, Aoi, Higashi-ku, Nagoya, Aichi Japan; 2 Graduate School of Public Health, St. Luke’s International University, Tokyo, Japan; 3 Australian Research Centre in Sex, Health and Society, La Trobe University, Melbourne, Australia; 4 Faculty of Health Science, Osaka Aoyama University, Osaka, Japan

**Keywords:** Help-seeking, Emotional support, Gay, Bisexual men, Sexual orientation concerns, Coming-out, Japan

## Abstract

**Objective:**

This study investigates Japanese gay and bisexual men’s experiences of seeking help for emotional support from others regarding their sexual orientation concerns. It examines the relationship between their help-seeking and presence of gay and bisexual peers, duration between questioning their sexual orientation and accepting it, and experience of coming out to family members by multiple logistic regression analysis.

**Results:**

We conducted a cross-sectional survey using a self-reported paper questionnaire. There were 360 valid responses. Eighty-two respondents (22.8%) had experience of help-seeking for emotional support, and this was associated with age, occupation, presence of gay/bisexual friends, and experience of coming out to family members about their sexual orientation. Respondents sought the most help from their male friends (70.0%), followed by female friends (25.0%), mothers (17.5%), and the Internet (16.3%). Even after controlling for age and occupation, experience of help-seeking for emotional support was higher among participants who had gay/bisexual friends when they were aware of their sexual orientation, took < 1 year from questioning to realizing their sexual orientation, and had come out to their family about their sexual orientation.

**Supplementary Information:**

The online version contains supplementary material available at 10.1186/s13104-024-06776-x.

## Introduction

In Japan, gay and bisexual men have poorer mental health than their heterosexual counterparts, with 31.0% reporting experiencing suicidal thoughts and 15.0% attempting suicide in their lifetimes. Gay and bisexual men are most at risk for suicide during their middle- and high-school years when first questioning their sexual orientation [1–3].

LGBT awareness is beginning to spread in Japan [4]; however, discrimination regarding sexual orientation remains unprotected by law, and many gay and bisexual men have difficulty accepting their sexual orientation [5, 6]. Longer duration until accepting their sexual orientation can elevates suicidal risk [7]. In Japan, it takes four years for gay and bisexual men to realize their sexual orientation after questioning it [6, 8] which is longer than in other countries [9].

For sexual minorities, schools are important places, and it is strongly recommended by the government that schools provide an environment in which lesbian, gay, bisexual, and transgender (LGBT) students feel comfortable seeking help for their sexual orientation-related concerns [10, 11]. A previous study in Japan found that 39.9% of school nurses were consulted by LGBT students for concerns related to sexual orientation [12]. Most nurses, the majority being female, were less inclined to report gay students among LGBT students consulting regarding their sexuality [12, 13]. Against this background, support from peers is important in facilitating smooth sexuality self-identification and protecting against mental health problems [7, 14–18]. However, no studies have examined how many and to whom gay and bisexual men have sought help regarding their sexual orientation concerns when they started questioning their sexual orientation they sought support in Japan.

Hiding sexuality from family members is a psychological burden for gay and bisexual men [6, 11, 19, 20]. Although the majority of LGBT individuals report wanting to come out their sexuality to family members, only 14.7% came out [21, 22]. It is possible that seeking help for emotional support from others help these individuals become more accepting and easier to come out to family members [9, 16, 23], but this association remains uninvestigated in Japan.

It has been reported that majority of gay and bisexual men who sought help for mental support did not disclose their sexuality for fear of a reaction [21, 24]. Studies have shown that people are more likely to seek support from people of the same gender as themselves, and those with more opportunities to interact with peers of the same sexuality are more likely to seek support [24]. However, few studies have also examined the effectiveness of peers, friends and family as sources for help [25].

Thus, this study aimed to (1) clarify Japanese gay and bisexual men’s experiences of seeking help for emotional support from others regarding their sexual orientation concerns, and (2) determine the relationship between the experience of seeking help for emotional support and presence of gay and bisexual peers, duration between questioning their sexual orientation and realizing it, and experience of coming out to family members about their sexual orientation.

## Methods

### Study design

We conducted a cross-sectional survey in 2016 at the LGBT festival in Aichi Prefecture. Participants were requested to complete an anonymous self-reported paper and pencil questionnaire. Eligibility criteria were being male, over 20 years old, being able to read and write Japanese. Trained staff members explained the eligibility criteria and invited participation. All participants provided verbal and written informed consent. Drinks and snacks were distributed to participants as a reward (approximately 1 USD in value). Ethics approval was granted by the Graduate School of Nursing at Nagoya City University (No. 15002-2).

The questionnaire included the following items: (1) background, (2) years of started questioning and realized their sexual orientation, (3) experiences of seeking help for emotional support regarding sexual orientation concerns, (4) the individual from whom they sought help for emotional support, (5) presence of gay or bisexual male friends when you were aware of your sexual orientation, and (6) disclosure of sexual orientation to family members.

Regarding item 1) background, it includes age, residence, gender, occupation, annual salary, and self-reported sexual orientation.

Regarding item 2), participants were asked about the age when they began questioning whether they might be attracted to men and when they realized their sexual orientation. The gap between these two points was calculated. Based on previous research, this number of years was assumed to be between 1 and 10 years [8]. Following discussions with two gay-led community organizations: Angel Life Nagoya and MASH Osaka, three categories were established: less than one year, (optimal); 2–5 years; and more than five years, (considered a long time).

Regarding item 3), participants were asked about experiences of seeking help for emotional support regarding sexual orientation concerns. Response options were: 1 person, 2 or more, or did not seek help from anyone.

Regarding item 4), participants were requested to select from 11 options: friends of the same sex, friends of the opposite sex, mother, father, brothers, sisters, colleagues, Internet/website, counselor, schoolteacher, and support group.

Regarding item 5), participants were asked if they have any friends or acquaintances who were gay, bisexual, or transgender, when they realized their sexuality.

Regarding item 6), respondents were asked if they had disclosed to their family members about their sexuality. Response options were yes or no.

Questionnaire items were developed only for this study and pretested with 10 members from two gay-led community-based organizations. The English version of the questionnaire is presented in Appendix A.

### Statistical analysis

We first examined the correlates of seeking help for emotional support regarding concerns with sexual orientation. These involved χ2 tests conducted independently for background variables and three predictor variables. Outcome variable was seeking help for emotional support regarding concerns with sexual orientation. Subsequently, we conducted multivariate binary logistic regression analysis (method: forced entry) assessing associations between each of three predictor variables and outcome variable while adjusting for background variables showed significance in χ^2^ tests. All statistical tests were conducted using IBM SPSS Statistics ver.22.

## Results

In total, 515 questionnaires were distributed and 505 were collected (98.1% collection rate). The 360 respondents (71.2%) who indicated that they identified as “male” and “gay” or “bisexual,” who were over 20 years old, and responded regarding an experience of seeking help regarding their sexual orientation concerns, were included in this study.

The average age was 31.2 years (SD = 7.8). The average time from questioning to realizing one’s sexual orientation was 4.2 years (SD = 4.2).

### Factors associated with experience seeking help regarding concerns with sexual orientation

Among the 360 respondents, 22.8% (*N* = 82) had experience with seeking help for emotional support regarding concerns with sexual orientation. Participants were divided into two groups based on whether they had sought help. Participants’ demographics, predictors, and experiences of seeking help for emotional support are shown in Table 1. Age (*p* = 0.024), occupation (*p* = 0.009), presence of gay/bisexual friends when they were aware of their sexual orientation (*p* < 0.001), and experience of coming out to family members about their sexual orientation (*p* = 0.018) were associated with experience of talking to someone about their sexual health concerns.

**Table 1:** Demographic factors and predictors by the experience of seeking help regarding concerns with sexual orientation

	Experience of seeking help for emotional support regarding concerns about sexual orientation						
	Yes (*n* = 82, 22.8%)		No (*n* = 278, 77.2%)		All		*p*
	n^1)^	%	n^1)^	%	n^1)^	%	
**Demographics**							
Age							
20–24	26	33.8%	52	19.3%	78	22.5%	0.024
25–34 years old	32	41.6%	126	46.8%	158	45.7%	
35 + years	19	24.7%	91	33.8%	110	31.8%	
Residence							
Tokai, Hokuriku	57	69.5%	201	72.3%	258	71.7%	0.438
Kinki	13	15.9%	50	18.0%	63	17.5%	
Other areas	12	14.6%	27	9.7%	39	10.8%	
Occupation							
Full-time, Manager	52	63.4%	218	78.4%	270	75.0%	0.009
Student, part-time, others	30	36.6%	60	21.6%	90	25.0%	
Yearly salary							
Less than 18,000 USD	15	18.3%	49	18.0%	64	18.1%	0.285
18,000–36,000 USD	38	46.3%	150	55.1%	188	53.1%	
Over 36,000 USD	29	35.4%	73	26.8%	102	28.8%	
Sexual orientation							
Gay	71	86.6%	251	90.3%	322	89.4%	0.412
Bisexual	11	13.4%	27	9.7%	38	10.6%	
**Predictors**							
1. Presence of gay/bisexual friends when you were aware of sexual orientation							
Yes	54	66.7%	39	14.2%	93	26.1%	< 0.001
No	27	33.1%	236	85.8%	263	73.9%	
2. Duration from questioning to realizing your sexual orientation							
Within 1 year	24	35.3%	68	25.7%	92	27.6%	0.080
2–4 years	30	44.1%	100	37.7%	130	39.0%	
More than 5 years	14	20.6%	97	36.6%	111	33.3%	
3. Experience of talking to family about your sexual orientation							
Yes	32	39.5%	70	25.6%	102	28.8%	0.018
No	49	60.5%	203	74.4%	252	71.2%	

^1)^The total number of items differs because missing values were excluded from the analysis

### Individuals from whom help was sought regarding sexual orientation concerns

**Figure 1:**
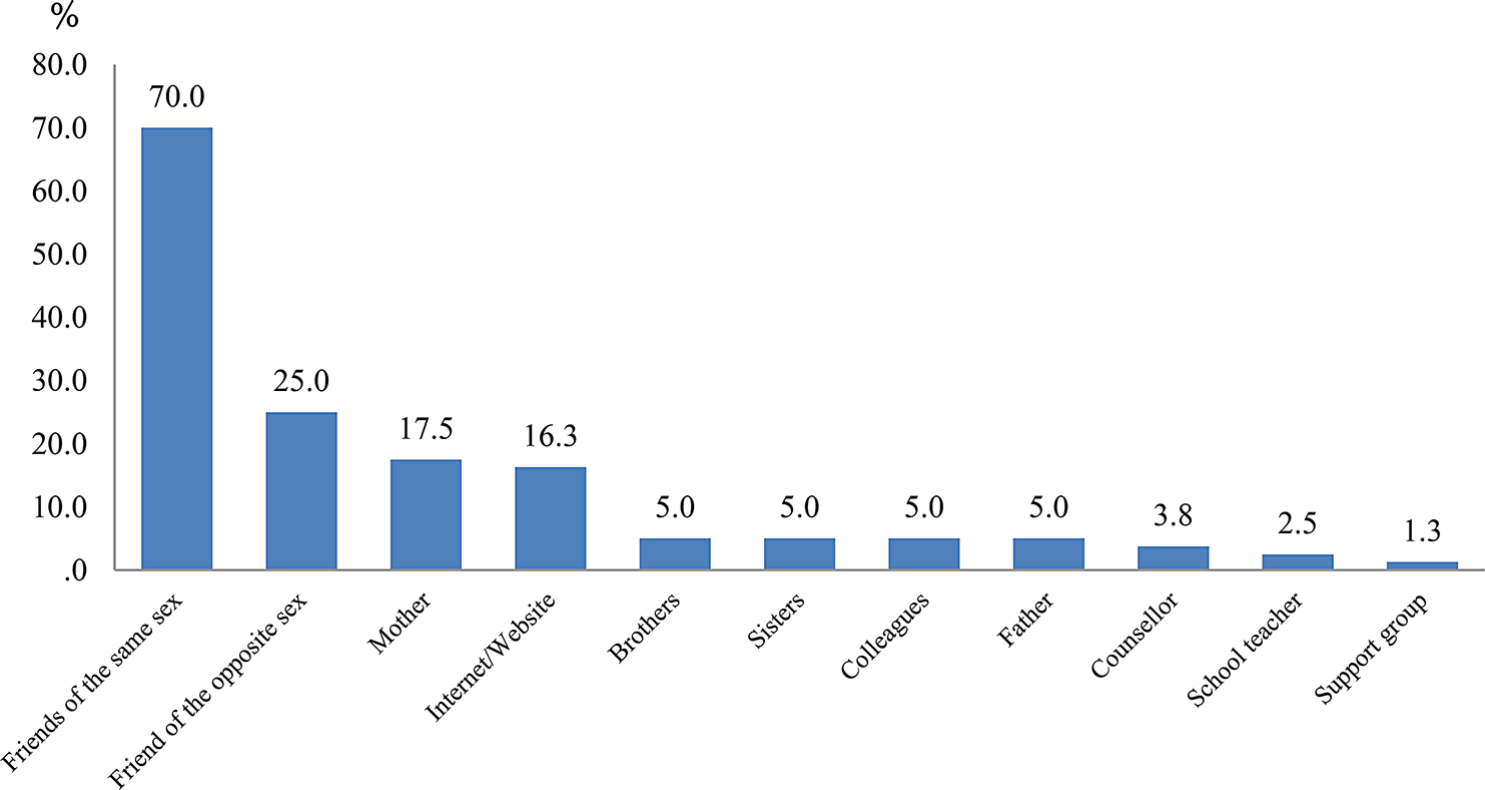
Persons from whom you sought help on your sexual orientation concerns* (Multiple answers, *N* = 80) *Restricted to only those from whom you sought help on your sexual orientation concerns

Gay and bisexual men sought the most help from friends of the same sex (70.0%), followed by friends of the opposite sex (25.0%), mothers (17.5%), and the Internet (16.3%). Other individuals consulted had a utilization rate of < 10%. These results are presented in Fig. 1.

### Associated factors for the experience of seeking help regarding concerns about sexual orientation

The multivariate model results are presented in Table 2. The results showed that the experience of seeking help for emotional support regarding concerns with sexual orientation was higher among participants who had gay/bisexual friends when they were aware of their sexual orientation, compared with those who did not (AOR, 12.13), took < 1 year to realize their sexual orientation compared with those who took > 5 years (within 1 year: AOR, 2.48), and had come out to their family about their sexual orientation compared with those who had not (AOR, 1.77).

**Table 2:** Association between predictors and seeking help for emotional support regarding concerns with sexual orientation

	OR	AOR^1)^
1. Presence of gay/bisexual friends when you were aware of sexual orientation^2)^		
Yes	12.1(6.83–21.50)	12.13(6.69–21.97) ***
No	ref	ref
2. Duration from questioning to realizing your sexual orientation^2)^		
Within 1 year	2.45(1.18–5.10)	2.48(1.14–5.41) *
2–4 years	2.08(1.04–4.16)	1.93(0.92–4.08)
More than 5 years	ref	ref
3. Experience of coming out to your family about your sexual orientation^2)^		
Yes	1.89(1.12–3.19)	1.77(1.03–3.06) *
No	ref	ref

1) Each AOR was calculated separately for each predictive variable

2) Forcedly controlled with age and occupation

* *P* < 0.05, ** *P* < 0.005, *** *P* < 0.001

## Discussion

Of all respondents, approximately one-fifth (22.8%) had sought help from others regarding their concerns about sexual orientation.

Although numerous studies highlight the poor mental health of gay and bisexual men [1, 6, 7, 15, 16, 26, 27], there are significantly fewer studies on the help-seeking behavior of these men, and no previous studies have focused on the situation in Japan, which is an academic concern [28]. Although previous research shows that 23.0-27.1% of LGBT individuals who have experienced suicide attempts previously sought help from someone else [2, 29, 30], there is no research targeting gay and bisexual men in Japan. The fact that 77.2% of participants in this study did not seek help for emotional support is concerning, given the high rate of suicide attempts among gay and bisexual men. Overseas, limited opportunities to meet peers of the same sexuality and the fear of disclosing their sexual orientation have been identified as barriers to help seeking behaviors [24, 28, 31]. In Japan, it is crucial to identify and address barriers and facilitators influencing help seeking behaviors. Recognizing that a significant proportion of sexual minorities are unable to seek help underscores the need to create an environment that fosters accessibility for young gay and bisexual men seeking support.

In this study, among those who sought help for emotional support, same-sex friends were the most common source of advice or support (70.0%), followed by opposite-gender friends (25.0%). Comparatively, counselors and teachers were much lower; 3.8% and 2.5%, respectively. This finding that friends are a more important source of advice than professionals is important and concurs with that of previous studies in other countries [2, 15, 32], which stresses the importance of support from friends. It is therefore necessary for society as a whole—and not merely clinicians who provide psychological support or specialists who work with youth—to deepen their understanding of sexual minorities [7].

Notwithstanding controlling for age and occupation, the results showed a significant association between the experience of seeking help for emotional support and presence of gay/bisexual friends, a shorter time until realizing their sexual orientation, and experience of coming out to family members.

Having gay and bisexual male friends may create more opportunities for discussing sexuality-related concerns. It may also make seeking help easier when they have serious concerns. Japan has five community centers providing consultations and support for gay and bisexual men mainly focusing on HIV prevention, which are managed by the Ministry of Health [33]. It is crucial to educate young people about resources that can provide support for sexuality-related concerns. Introducing them to such centers and providing opportunities to connect with peers may help them seek emotional support when they have problems [34].

Shorter duration from questioning to realizing sexual orientation was associated to having experiences of coming out to family members. Research on gay and bisexual youth conducted abroad has shown that support from friends and family is associated with lower mental distress and facilitates easier acceptance of one’s sexual orientation [9, 11, 31, 34]. Future research is required on what types of support, beyond help-seeking for emotional support, can make disclosing one’s sexual orientation to their parents easier.

This study’s strength is that it was conducted in Japan, where sexual minorities’ visibility lags behind [4, 35] and studies on their help-seeking behavior is sparse. The fact that their help-seeking behavior is associated with having friends of the same sexuality, a shorter period of conflict on their sexuality and coming out to their families, highlights the need for creating an environment in which sexual minorities feel comfortable seeking help.

In conclusion, this study observed that help-seeking for emotional support regarding concerns about sexual orientation positively associated with the presence of gay/bisexual friends when individuals were aware of their sexual orientation, a duration of < 1 year between questioning and realizing their sexual orientation, and experience of coming out to family about their sexual orientation. Additionally, same-sex friends were the most commonly consulted, suggesting the importance of same-sex peers’ understanding of sexual orientation concerns.

## Limitations

Only recently have social awareness and interest in sexual minorities increased in Japan. The majority of respondents in this survey were in their 30s and 40s, which may not accurately reflect the reality of sexual orientation-related consultations among young people today. In addition, this survey was conducted at an event with many LGBT participants who are more sociable or more likely to disclose their sexualities. Therefore, the results may not apply to hidden gay and bisexual men. This study was conducted in 2016, however, more recent research in Japan does not exist. Therefore, these results provide valuable insights.

## Electronic supplementary material

Below is the link to the electronic supplementary material.

Supplementary Material 1

## Data Availability

The datasets used and/or analyzed during the current study are available from the corresponding author upon reasonable request.

## Abbreviations

AOR: Adjusted odds ratios
LGBT: Lesbian, Gay, Bisexual, and Transgender
