# Supplementary material for: Factors associated with help-seeking regarding sexual orientation concerns among Japanese gay and bisexual men: results from a cross-sectional survey
Source: BMC Res Notes. 2024 Apr 23;17:117. doi: 10.1186/s13104-024-06776-x (PMC11040953; doi:10.1186/s13104-024-06776-x)
Supplement: Supplementary file 1 — Supplementary Material 1 [file 13104_2024_6776_MOESM1_ESM.docx]

**Questionnaire**

Q1 Is this your first time answering this questionnaire?

1. First time
2. ( ) times

Q2 Where do you live in Japan?

( 　　　 ) prefecture

Q3 　What is your gender?

1. Male
2. Female
3. Other ( 　　　 )

Q4 How old are you?

( 　　　 ) years old

Q5 What is your current occupation?

1. Student
2. Part-time job
3. Full-time
4. Part-time
5. Manager
6. Other ( 　　　　　 )

Q6 What is your annual income? (If you have no income, please tick □1)

1. 0 to 2 million yen
2. 2-3 million yen
3. 3-4 million yen
4. 4-5 million yen
5. 5-6 million yen
6. 6-7 million yen
7. 7-8 million yen
8. 8 million yen and up
9. Other ( )

Q7 Which of the following applies to you? (Tick only one)

1. Gay →go to Q8
2. Bisexual →go to Q8
3. Transgender → go to Q8
4. Heterosexual → go to Q11
5. Don't know, don't want to decide, other ( ) → go to Q11

Q8 Please tell us how old you were when you became aware of your sexuality.

Please tell us the age you started feeling "I might be gay, or I might like male..." or "I might be attracted to the same sex guy."

( ) years old

Q9 Please tell us the age when you became aware of your sexuality.

Please tell us the age when you really **assured** that you were gay or you were attracted to the same sex guy.

( ) years old

Q10 When you became somewhat aware of your sexuality, did you have any friends or acquaintances who were gay, bisexual, or transgender?

1. Yes
2. No

Q11 Around the time you became somewhat aware of your sexuality, did you talk to others and ask for advice about your sexual orientation concerns? If yes, how many people did you discuss it with?

1. 1 person
2. 2 or more
3. I did not or consult with anyone about it → go to Q12

Q11-1 We would like to ask those who answered 1 or 2 or more people in Q11.

Whom did you talk to and ask for advice about your sexual orientation concerns?　(Tick all that apply)

1. Father
2. Mother
3. Brothers
4. Sisters
5. Friend of the same sex
6. Friend of the opposite sex
7. School teacher
8. School nurse
9. Counselor
10. Colleagues
11. Internet/website
12. Support group
13. Public health nurse
14. Other ( )

Q18 Have you ever talked to any of your family members about your sexuality?

1. Yes
2. No

Thank you for your cooperation.
